# Developing a Text Messaging Intervention to Increase Uptake of the Screening and Treatment for Anxiety and Depression Program Among Community College Students: Formative Study Using a Human-Centered Design Approach

**DOI:** 10.2196/84640

**Published:** 2026-07-21

**Authors:** Tamar Kodish, Francisco Reinosa Segovia, Pamela V Pichon, Kate Wolitzky-Taylor, Michelle G Craske, David C Mohr, Denise A Chavira

**Affiliations:** 1 Department of Psychology and Neuroscience, University of Colorado Boulder, Boulder, CO, United States; 2 Department of Psychiatry and Biobehavioral Sciences, University of California Los Angeles, Los Angeles, CA, United States; 3 Department of Psychology, University of California Los Angeles, 1285 Franz Hall, PO Box 951563, Los Angeles, CA, 90095, United States, 1 310-825-8466; 4 Department of Preventive Medicine, Center for Behavioral Intervention Technologies, Northwestern University, Chicago, IL, United States

**Keywords:** digital mental health, co-design, text message intervention, implementation science, community college, treatment barriers

## Abstract

**Background:**

Community college (CC) students face significant mental health concerns but are unlikely to receive treatment. Barriers to mental health service uptake among CC students have been delineated, but few studies have identified strategies to improve uptake. Text messaging has been used to address engagement barriers to mental health services among adolescents and adults, but little research has explored this strategy for CC students.

**Objective:**

The goal of this study was to partner with CC students to co-design and conduct pilot usability testing of a text messaging intervention to address barriers and increase uptake of a mental health screening and treatment program, called Screening and Treatment for Anxiety and Depression (STAND), offered to CC students.

**Methods:**

We conducted 2 parallel sets of 4 co-design focus groups with CC students who had varying levels of engagement with STAND. We used rapid qualitative analysis to extract key themes, create text message prototypes and refine them, and present updated prototypes to gather feedback across workshops. We also assessed six usability factors on a 5-point Likert scale: satisfaction, helpfulness, attractiveness, readability, comprehension, and likelihood of getting started with STAND after receiving texts.

**Results:**

Key themes emerged about perceptions of texting, barriers to STAND, a basic framework for the text message intervention, feedback about the format of messages, and feedback about the content of messages. Students expressed positive regard for text messaging and general agreement on key barriers to STAND. Students codeveloped a framework for the intervention, including (1) delivering introductory texts to engage students in the text messages, (2) providing a personalized approach for students to select barriers most salient for them, and (3) delivering tailored content designed by students to address each barrier. Across workshops, several themes emerged with regard to how messages should be formatted and delivered, including the following: use short messages; use not too many messages; use relevant language; use images, memes, and short videos; and make messages “human-like.” Themes related to the content of messages included the following: reminders that you are not alone, knowledge that STAND has worked for other students, expressing understanding of student context and stressors, and providing an option to speak to a team member. Mean ratings on usability factors ranged from 3.88 (SD 0.64) to 4.25 (SD 0.46).

**Conclusions:**

This study describes a process for co-designing a text messaging mental health engagement intervention with CC students that is grounded in a human-centered design approach. Further research is needed to rigorously test this intervention and make iterative refinements to improve response and effectiveness.

## Introduction

### Background

Community college (CC) students in the United States have significant mental health needs, but they are much less likely to receive treatment relative to their four-year university counterparts [1]. Given gaps in mental health treatment access and usage for CC students, understanding barriers and increasing engagement in mental health services for this population is a critical objective shared among students, administrators, and policymakers [1]. Compared to four-year university students, CC students are more likely to identify as racial or ethnic minorities, be first-generation college students, come from lower–socioeconomic status backgrounds, and, on average, face more contextual stressors such as food insecurity and houselessness [2]. System-level barriers, such as insufficient mental health service infrastructure, shortages of campus providers, lack of culturally responsive services, and cost of treatment, are major impediments to service receipt among CC students [1,3,4]. Individual level barriers to treatment have also been documented, including insufficient time to seek services, beliefs that treatment is not needed, limited knowledge of mental health resources, and stigma [5,6]. Recent campus-wide initiatives to increase access to mental health screening and treatment have been developed and implemented to close these gaps and enhance treatment quality and access for underserved CC students [7,8].

One such program is the Screening and Treatment for Anxiety and Depression (STAND) program, a free, evidence-based tiered system of mental health screening and treatment created for CC students [7-9]. Within STAND, participants complete an online screening for mental health symptoms and are matched with a level of care that best meets their needs, ranging from (1) a self-guided online wellness program, to (2) an online cognitive behavioral therapy with peer coach support, to (3) clinician delivered evidence-based psychological treatments and psychiatric care (for more detailed description of STAND, see Wen et al, 2023 [8]). Although STAND addresses many common barriers to care, making services free, accessible, and convenient for students, preliminary findings suggest suboptimal rates of treatment initiation. In the four-year university context, findings indicated that just 8% of students were eligible for STAND care after screening initiated STAND services [9]. These rates of treatment conversion are consistent with other studies of student mental health interventions [10]. Outside of college student populations, the literature similarly underscores low rates of user uptake of digital mental health interventions [11,12]. Despite low rates of treatment uptake, most research on digital mental health engagement has focused on strategies to retain users in digital interventions once they are already enrolled, without emphasizing how to help individuals “get their foot in the door” [13-15]. While retention is a major engagement objective, experts in behavioral health technology suggest that adoption, which is defined as one’s intention, decision, or initiation of a program, is another core, but understudied, measure of implementation success [16]. Development of novel strategies with the potential to improve initial uptake of mental health screening and treatment is needed to address the mental health crisis among CC students.

### Texting as Engagement Strategy

To date, strategies with the potential to improve uptake of mental health services among underserved CC students are not well defined or understood. One strategy that has been used in other populations (eg, adolescents and caregivers) to improve uptake and engagement in mental health services is text messaging [17]. Text messaging interventions that address barriers to treatment usage have been found to be acceptable, feasible, and effective in improving mental health service initiation among adolescent and young adult samples [17,18]. For example, one study designed a text message intervention to address cognitive barriers and increase mental health service usage for adolescents who screened positive for depression in primary care [17]. The intervention focused on addressing concerns and bolstering the benefits of mental health care through an interactive, psychoeducation-based SMS system. Findings revealed that overall, adolescents and their caregivers were satisfied with this program, and rates of mental health service usage were higher than rates reported in the literature, with 52% of those who received text messages reporting that they attended a mental health appointment. While text messaging microinterventions have been developed to increase mental health service engagement for other populations, to our knowledge, no such interventions have been specifically designed for CC students. In addition, while these interventions hold significant appeal, their reach may be limited for individuals without consistent access to phones, and rates of engagement with text-based messages may be suboptimal.

Text-based approaches represent an especially promising pathway to promoting uptake of mental health services for CC students. Text messaging is a convenient, accessible method of communication that fits seamlessly into the day-to-day lives of CC students. CC students also report a high willingness to use online mental health services [19]. This comfort with technology may translate to openness to using text-based supports. Texting may be an especially effective method for reaching CC students because they report frequently juggling multiple roles and responsibilities, leading to high demands on their time and limited capacity to engage in treatment. Prior work also highlights the importance of tailoring text messages to increase fit with student preferences [20]. One study examining text messages to reduce alcohol use among CC students revealed that messages that were selected by students (rather than investigators or at random) were perceived as more useful by students [21].

### Human-Centered Design Approaches

Designing new interventions in partnership with end users is needed to enhance the feasibility and acceptability of these interventions [4,22]. Centering user knowledge of their own communities, goals, and perspectives throughout the entire development and implementation process is viewed as essential to the successful design of mental health interventions [23]. Accordingly, principles of human or user-centered design (HCD) are increasingly used to guide the development of mental health interventions themselves, as well as the implementation strategies that support their use [23-25]. HCD is an approach to developing products that is grounded in collecting information from individuals for whom and in settings in which products will ultimately be used [26,27]. HCD models also highlight the iterative nature of data collection and intervention development, emphasizing the need for continuous integration of user and stakeholder feedback to produce the most usable and engaging products [23]. Indeed, co-designing interventions with community stakeholders was rated as the single most important strategy for improving engagement in digital mental health interventions among CC students of color by experts, further emphasizing the salience of these principles for this specific population [4].

Recently, scholars have integrated HCD/user-centered design elements with an implementation science framework to create the Discover, Design, Build, and Test (DDBT) model [24,25]. This framework is intended to guide the development of interventions and implementation strategies to be deployed in real-world contexts with resource constraints. The discover phase is characterized by identifying stakeholder needs and points of view, understanding the implementation landscape, and current problems. The design and build phases involve synthesizing findings and insights from the discover phase to codevelop solutions with community stakeholders, transform these solutions into low-fidelity prototypes, and collaboratively engage in an iterative process of testing and refining these solutions. In the test phase, pilot testing of prototypes is conducted to evaluate feasibility, acceptability, and preliminary outcomes in a real-world context.

### Study Goals

This study seeks to narrow several major gaps identified in the literature. First, this project brings attention to a high mental health–need, low-resource, and underserved population of CC students, who have been frequently overlooked in the literature. Second, this project leverages technology to develop an engagement-focused intervention that can fit into the fabric of students’ lives. Third, this project uses human-centered design methods to ensure CC student voices are amplified and centered throughout the intervention development process. Last, this project focuses on designing an intervention that attempts to address dropout from services rather than engagement once individuals are already enrolled, homing in on a key target that has been relatively understudied. This paper describes the application of the DDBT model to design, build, and pilot-test a text messaging microintervention with the intention to increase uptake of the STAND program, a tiered system of mental health care, for CC students. The objectives of this study were to (1) design and build a text messaging intervention to increase STAND uptake among CC students and (2) pilot-test the intervention with a small group of students to explore the acceptability and feasibility of the intervention.

## Methods

### Ethical Considerations

This study was approved by the University of California, Los Angeles Institutional Review Board (IRB#23‐000138). Informed consent was obtained from all participants before data collection. Privacy and confidentiality were maintained through the deidentification of all data. Participants were compensated with a US $50 gift card for their participation in each group.

### Study Context

This study was conducted within the University of California Los Angeles STAND for Community Colleges Innovation Center (P50MH126337). The main goal of the Center is to implement the STAND system of care in a large CC, East Los Angeles College (ELAC), and develop a template for scaling up the STAND system to CCs across the state. ELAC is an accredited CC that offers both academic transfer courses, preparing students for transfer to a four-year institution, as well as occupational programs, preparing students for careers in two years or less. ELAC serves ≈36,000 students and awards ≈10,000 degrees or certificates each year, making it the largest CC in California. As a result of its size and location, ELAC educates a diverse, majority Latinx student body, with students from throughout the city and the world. These students come from a variety of backgrounds with unique perspectives and needs, often underrepresented among college populations, including undocumented, veteran, single-parent, formerly incarcerated, and disabled students. Students come from a variety of economic backgrounds, with a large proportion facing food or housing insecurity or homelessness.

Preliminary work for this study focused on understanding factors that encourage and discourage STAND engagement among diverse students. Within this previous project, we engaged in the “discover” stage of our process by conducting a set of focus groups to identify key barriers and facilitators to uptake STAND among CC students, described in detail elsewhere [28]. Analyses of these data led to the identification of the key barriers and facilitators to STAND services among ELAC students. Key barriers included the following: limited mental health knowledge or literacy, negative beliefs about mental health and treatment, logistical barriers including insufficient time and lack of privacy, technology-related concerns, lack of perceived need for STAND, and insufficient knowledge about STAND. After discovering core barriers to STAND through this prior series of focus groups, this study used a co-design workshop approach to design, build, and conduct small-scale, preliminary pilot usability testing of a text message intervention that addresses student barriers by providing SMS-based text messages of support and information to overcome these obstacles.

### Participants and Design

**Figure 1.**
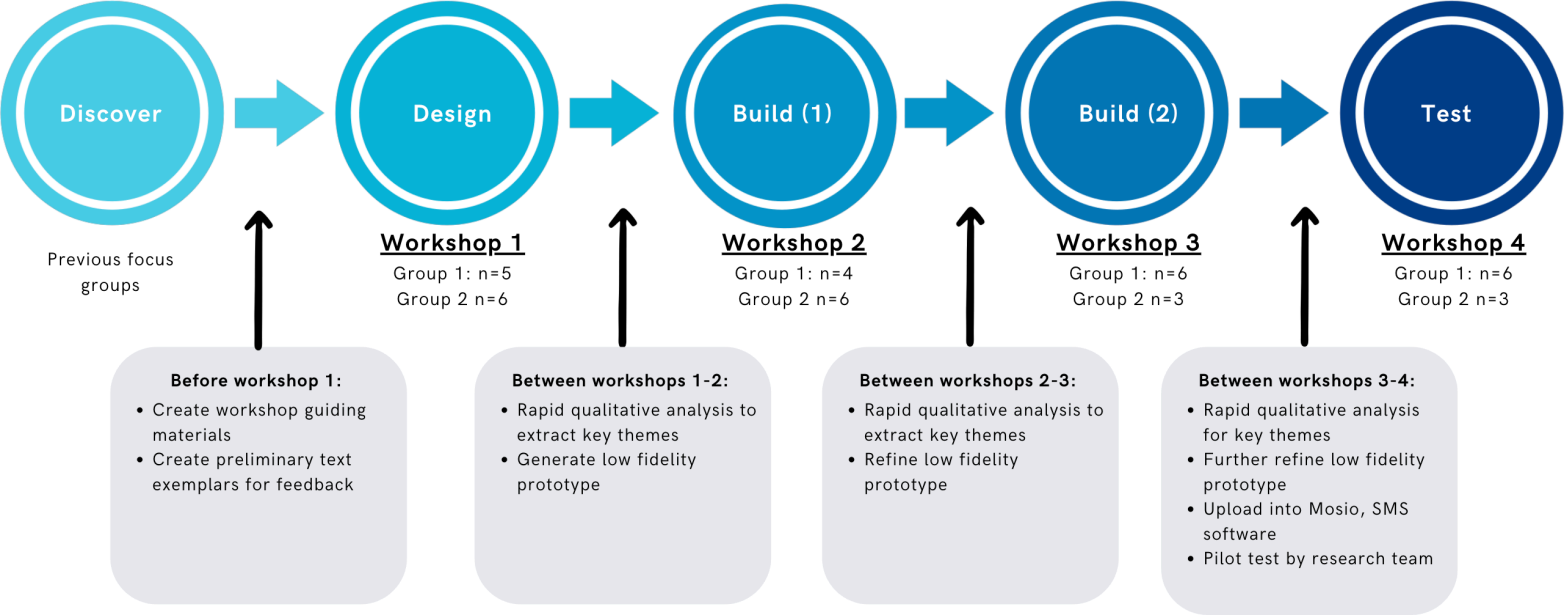
Co-design process/DDBT model application. DDBT: Discover, Design, Build, and Test.

We conducted two parallel sets of four co-design workshops with CC students with the goal of designing, building, and conducting preliminary usability pilot testing of a text messaging intervention to address barriers to STAND. Each workshop stage (1-4) involved two separate groups, aligned with best practice recommendations for focus groups. Workshops were facilitated by authors TK, FRS, and PVP. For a visual representation of this study’s design, see Figure 1.

Workshops 1‐3 were a sequential workshop series in which participants were invited to participate in 3 sequential groups. Eligible participants for the workshops 1‐3 series were drawn from a previous study that recruited students with varying levels of STAND engagement, including students with no STAND contact, students who started but stopped the STAND screening survey, and students who completed STAND screening and attended orientation. As the previous study focused on identifying barriers and facilitators to STAND, we recruited from within this pool for this study to extend previous “discover” stage work into the design, build, and test stages. For this study, students who participated in previous groups and agreed to be contacted about future research were recruited by phone outreach and were invited to attend a series of three workshops (1 design workshop and 2 build workshops). We invited participants to engage in all three workshops from the beginning to enhance continuity and facilitate multiple opportunities to provide feedback, as recommended in previous co-design processes.

A fourth set of workshops (test) was conducted with a new set of students for pilot testing and continued generation of feedback from a novel user audience. These students included those who (1) completed STAND screening, (2) had severe anxiety and/or depression scores, and (3) agreed to be contacted by the study team for future research. We selected students from this group for the final workshops to ensure that students with higher levels of mental health need were represented in the co-design process and that the content we developed in earlier stages was appropriate for students experiencing more severe symptomology.

Across workshops, participants were compensated US $50 per group. Sessions were on average 72 (SD 12.8) minutes long. All focus groups were conducted remotely using HIPAA (Health Insurance Portability and Accountability Act) compliant videoconferencing software. While we opted to conduct workshop groups remotely to optimize flexibility and accommodate scheduling constraints, the remote workshop format may have limited or impacted the level of engagement among participants.

The goal of workshop 1 was to explore openness to a text messaging intervention and begin designing a preliminary prototype for this intervention. Subgoals included the following: (1) introducing and generating feedback on the text messaging microintervention concept, (2) finalizing and refining the list of key barriers to STAND, and (3) beginning the rapid prototyping process. Students were first shown the list of barriers noted above, drawn from previous focus groups, and asked to refine, add, and remove any essential barriers. Then, students viewed a brief research-based presentation about text messaging interventions and responded to open-ended prompts to generate their perceptions, reactions, and thoughts about this format of intervention delivery for themselves and their peers. Last, students were shown each of the barriers and asked questions such as “What would you need to hear to overcome this concern and get started with STAND?” They were also provided with several examples of responses to support students in overcoming these barriers, including graphics, videos, images, memes, and text messages, and asked to share their opinions and ideas about these responses.

The goals of workshop 2 were to begin building the text messaging intervention through (1) presenting preliminary prototypes developed from the first workshop and (2) attaining student feedback and revisions to refine the prototype draft. The goal of workshop 3 was to generate additional feedback and finalize a working prototype of the text messaging intervention. Rapid prototypes were built using real text messaging templates to display the intervention in the most realistic format possible.

The goal of workshop 4 was to pilot-test the intervention with a novel group of student users to determine whether the intervention was acceptable to a group of stakeholders beyond the original co-design group. This approach of recruiting a new group of users for pilot testing has been implemented in previous DDBT studies (Jenness et al, 2022 [29]). In addition to user testing and qualitative feedback gathered within this workshop, we attained participant ratings on several usability domains. Items were drawn from a literature review on the usability of digital and texting interventions, including the following: satisfaction (overall, how satisfied are you with the content of text messages, 1=strongly disliked it to 5=strongly liked it), helpfulness (overall, how helpful did you find these text messages, 1=not at all helpful to 5=extremely helpful), attractiveness (overall, these messages attracted my attention, 1=strongly disagree to 5=strongly agree), readability (overall, these messages were easy to read, 1=strongly disagree to 5=strongly agree), comprehension (overall, these messages were easy to understand, 1=strongly disagree to 5=strongly agree), and goal attainment (if you were a student who received these messages, how likely would you be to get started with STAND? 1=very unlikely to 5=very likely). Participants were asked to bring their phones to the workshop, and with their approval, they were sent the text messages from our system and prompted to engage with the intervention on their own phones during the group, and provide both qualitative and quantitative feedback.

### Analyses

Focus group recordings were transcribed using auto-generated tools within the HIPAA-compliant videoconferencing software and reviewed for accuracy by a member of the research team. Rapid qualitative analysis (RQA) was used between each stage of workshops to (1) identify key themes and patterns, (2) integrate participant feedback into iterative prototypes, and (3) prepare presentation of new prototypes at subsequent workshops. RQA is a method used frequently in implementation research that emphasizes efficiency and speed in the data analysis process while maintaining rigor. It has been shown to be as effective as traditional qualitative analysis approaches [30]. We opted to use RQA because it is well-suited for the synthesis of semistructured focus group data and its focus on practice-oriented implementation research. Further, our process required quick and efficient analysis between co-design workshops in order to produce prototypes of the intervention to present to the next set of scheduled groups.

Per standard RQA procedures, two members of the research team used the co-design workshop group guides to develop templated transcript summary sheets with domains following from sections of each guide. The summary sheets were then used to extract key themes pertaining to the primary research questions and co-design process. Summary sheets extracting key themes arising in each group were completed independently and reviewed with two research team members during meetings between each round of workshops. The goal of these meetings was to review theme identification, refine and interpret key themes, resolve discrepancies in key themes, and attain consensus on summary sheets. Once consensus on key themes from each set of groups was met, the research team identified specific elements of the intervention to develop, refine, and present for feedback in the subsequent set of workshops. The specific language, images, and other contents of the text messages included in the intervention were repeatedly modified based on our analysis of participant feedback and input after viewing each set of prototypes. The research team worked collaboratively asynchronously and via weekly meetings between workshops to ensure that changes to the intervention prototypes reflected the qualitative themes extracted through the RQA process.

Throughout the iterative design process, we used several strategies to enhance trustworthiness in qualitative research, such as credibility, confirmability, and dependability. We addressed credibility and confirmability primarily through member checking, integrating into each workshop an overview of our RQA findings from the previous group to ensure that our findings were aligned with their experience of their input from the prior group. We asked open-ended questions after presenting our analysis to understand what we might be missing and add these data to our next round of analysis. We promoted dependability through a clear documentation of the process and steps and used the same sequence of procedures between each round of groups, including (1) summary sheet development, (2) independent review, (3) group review to resolve discrepancies and meet consensus, (4) clear actionable steps for refining prototypes based on analysis, and (5) internal review of updated prototypes before presenting to participants. We addressed reflexivity through explicit team debriefs in which we labeled and discussed our own identities, worldviews, and biases that might impact our interpretation of findings.

## Results

### Participants

A total of 19 students participated across all four workshops. Of these, 11 students were participants in the workshops 1‐3 series, and 8 participants participated in the workshop 4 groups. Of the 11 participants who participated in the workshop 1‐3 series, 7 (64%) participants attended all three workshops, while 4 participants missed one of the three workshops due to scheduling issues. None of the participants missed more than one workshop, so all had at least two opportunities to provide input for the design and build process, with the majority fully participating in all three workshops. For workshop 4, a total of 8 new students participated. Table 1 depicts the number of participants in each workshop group and information about the demographic makeup and level of STAND engagement within each group. The racial or ethnic breakdown of the overall (n=19) sample was 84.2% (16/19) Latinx, 5.2% (1/19) Asian American, 5.2% (1/19) Black or African American, and 5.2% (1/19) multiracial. The sample was 63.2% (12/19) female and 36.8% (7/19) male. The mean age of the sample was 27 (SD 5.9) years.

The following results are organized by workshop number and corresponding DDBT stage and include (1) themes arising in the rapid qualitative coding process, (2) illustrative quotes, (3) prototype illustrations, and (4) usability or acceptability rating outcomes. We also overview the activities of the research team between rounds to illustrate the implementation of our co-design process (Figure 1 for overview). Table 2 provides an overview of core topics covered in each workshop, and Table 3 provides an overview of the key themes and whether or not they emerged in each sequential set of groups.

**Table 1.** Sample demographics and STAND^a^ engagement levels.

	Students, N	Latinx, n (%)	STAND level of engagement, n (%)		
			No STAND participation	STAND screening started but not completed	STAND orientation completed
Workshop 1 (A)	5	5 (100)	2 (40)	0 (0)	3 (60)
Workshop 1 (B)	6	5 (83.3)	3 (50)	2 (33.3)	1 (16.7)
Workshop 2 (A)	4	3 (75)	3 (75)	0 (0)	1 (25)
Workshop 2 (B)	6	6 (100)	2 (33.3)	1 (16.7)	3 (50)
Workshop 3 (A)	6	6 (100)	3 (50)	2 (33.3)	1 (16.7)
Workshop 3 (B)	3	2 (67.7)	2 (67.7)	0 (0)	1 (33.3)
Workshop 4 (A)	4	3 (75)	0 (0)	1 (25)	3 (75)
Workshop 4 (B)	4	4 (100)	0 (0)	0 (0)	4 (100)

a STAND: Screening and Treatment for Anxiety and Depression.

**Table 2.** Core topics covered in workshops 1‐4.

Workshop	Core topics
Workshop 1	Review and finalize barriers Feedback on text message method Preliminary prototype development
Workshop 2	Feedback on themes from workshop 1 Rapid prototyping
Workshop 3	Rapid prototyping
Workshop 4	Pilot intervention Rate usability

**Table 3.** Key text message content and format themes.

Themes	Workshop 1	Workshop 2	Workshop 3	Workshop 4
Format				
Short, limited number of messages	✓			✓
Use of images, memes, and visuals	✓			✓
Use of videos	✓			
Response options and personalization		✓		✓
Include option to speak to a team member		✓		✓
Content				
Introduction	✓	✓	✓	✓
Personalized barriers approach	✓	✓	✓	✓

### Design: Workshop 1

Regarding the design of workshop 1, the results from the first co-design workshops were categorized across the three focus group guide sections: (1) regarding the barriers, refining and finalizing the list of key barriers to STAND; (2) regarding feedback on the text messaging method, generating student perspectives on using text messaging as an engagement strategy; and (3) regarding preliminary prototype development, beginning the co-design process.

### Workshop 1, Section 1: Finalizing Barriers

First, consensus about the key barriers emerged, and barriers were refined. Key barriers identified in previous “discover” focus groups included the following: (1) limited mental health knowledge or literacy, (2) negative beliefs about mental health and treatment, (3) logistical barriers including insufficient time and lack of privacy, (4) technology-related concerns, (5) lack of perceived need for STAND, and (6) insufficient knowledge about STAND. Across groups, students were shown these barriers and agreed that these were the most pertinent barriers to STAND. Students shared that logistical barriers should be split apart into “not enough time” and “concerns about privacy and confidentiality.” Students also felt that limited mental health knowledge or literacy could be collapsed with insufficient knowledge about STAND, given that our focus was explicitly on the STAND program. Although students identified some additional barriers through this process, no consensus about any additional barriers emerged across participants and groups. In this section of the workshops, students also recommended framing barriers using short “I” statements. A list of “I”-led barriers was developed from the preliminary barrier list (Multimedia Appendix 1).

### Workshop 1, Section 2: Feedback on Text Messaging Method

#### Overview

A variety of positive and accepting perspectives about the method of text messaging as an engagement strategy for CC students emerged. These themes included: casualness or ease, convenience and fit with busy student schedules, ability to respond quickly, reminders, capacity to address barriers.

#### Casualness or Ease

Participants shared that texting is a casual and easy way of communicating. For example, one participant shared:

*I feel like texting is a, it’s a better form of communication. I feel like it’s more casual and like you’re able to respond fast as opposed to like an email where you need to think it out more thoroughly, and I feel like texting is more casual and it’s easier. So, I feel like this is a good idea.*

#### Convenience or Fit With the Schedule

Students also emphasized that texting is aligned with their busy lives and schedules. One student noted:

*I actually think it’s a great idea that you guys are switching over to text messages, I’m more inclined to replying through text versus like email or like answering a phone call, especially right now that I’m in nursing school like I’m pretty busy*.

Another student said that texting can be helpful when students are not able to talk on the phone, sharing:

*I feel offering the text message would be like beneficial for the students, since maybe they can’t be on a call right now, but they can text you, or maybe they’re just not in the mood for a call, and texting would be beneficial for them.*

#### Quick Response

Students also shared that text messaging enables rapid responding, which they viewed positively. One student shared:

*Yeah, I think for myself, yeah, text messages would work because even though it would be automated, it would still be easier for me to read and just to click on the link and just get started. So, I feel like text message is a great idea, and it’s faster.*

Another shared, “So, it’s like a quick text is awesome…you know…‘Okay, cool, like, I’ll just reply, really quick.’”

#### Helpful Tool for Reminders

Students noted that using text messaging can be a useful reminder to prompt students to remember to engage in certain activities. Some students also reported experience using apps that included reminders in other aspects of their lives. For example, one student shared:

*I’m a religious person, and I have the Bible app and on the Bible app every single day, you know, maybe…maybe one time or 2 times a day, they send a message, “don’t forget to breathe, take a breath in, and out,” you know, just like little small reminders…some people receive it, and then they go, “Oh, let me remember! And then to take a breath, call myself down.” That would probably help.*

### Workshop 1, Section 3: Preliminary Prototype Design

Given the overall positive consensus about the use of text messaging to respond to barriers and promote uptake of STAND, the next section of the workshop began defining parameters and ideating concepts for this intervention. Several themes emerged about text message (1) format and (2) content across groups. We also explored key implementation questions, including who the target group for this intervention should be and at what time points they should receive messages. Students were shown various time points where the intervention could be delivered, and consensus emerged that the text messages should be sent to those who start the STAND screening process, but then either do not complete the screening, or complete it but then disengage and do not make it to the initial session. This “disengaged” group was identified because they represent individuals who expressed initial interest, but likely encountered barriers that stopped them from completing the STAND onboarding process and getting started.

Regarding format or delivery, several themes emerged with regard to how messages should be formatted and delivered, including (1) short and limited number of messages; (2) use of images, memes, and visuals; and (3) use of videos.

Regarding the format or delivery of short and limited number of messages, with regard to message format, students expressed that texts should be short and not too dense, citing that they would not be likely to read lengthy texts. One student suggested, “Personally, I have a very short attention span. It’s not good, but I have a very short attention span, so breaking down those into like one sentence messages it would be…that would be good.” Participants also stated that they did not want to be bombarded with messages, so there should not be too many messages. One student noted, “If the text messages are excessive, then like, I feel like some people would like, ignore them, and kind of just like, just yeah. Ignore them and not pay attention to them or respond.”

Regarding format or delivery of images, memes, or visuals, students expressed enthusiasm for images, memes, and visuals, noting these are “easier on the eyes” and “more appealing.” For example, one student noted:

*I think they’re good, to be honest. Especially like if you’re a visual learner, or if you’re like a visual person that you need to see something, I feel like sometimes by just looking at a picture it’s easier than reading like a whole long paragraph.*

However, students also shared concerns that images or memes might be misinterpreted or misunderstood, “I feel like some cons for the picture, some people might interpret it wrong, or like they won’t understand like what it means maybe….” Participants also expressed some concerns about technical issues related to receiving images or memes, for example:

*If it comes in that format where it doesn’t show the picture it more so just says, “Hey, download this PDF” no one’s going to, there’s going to be some people that will be skeptical and that will not open the PDF or that will just block you before even doing anything.*

Regarding the format or delivery of videos, students also expressed positive opinions about short videos, including video testimonials, recommending a “reel” “TikTok” format, but warned against videos that are too lengthy. For example, one student shared “So like, if it’s like a long video like three minutes like it’s not, it’s going to make me like not want to watch it, ‘cause it’s too long. So, I feel like the shorter the better.” Another student said: “If it’s more than 5 minutes I’ll probably be like, ‘oh okay, this is taking too long, you know, I’m going to click it out.’” Students also expressed that video testimonials from students who had previously participated in STAND might be useful. For example, one student stated, “I would like to hear like someone’s perspective that’s more my age or different age groups…different testimonies.”

Regarding content, we explored content for two potential arms of the text messaging intervention. First, we asked students how they might want to be introduced to the text messaging intervention or system. Students expressed desire for (1) an introduction that provides an overview of STAND via text and (2) a personalized approach that allows the recipient to select specific barriers that are most relevant for them, and then sends information that directly addresses the selected barrier.

Regarding content, introduction, students were asked how they would like this intervention to first be introduced via text. Themes extracted from their feedback included that an introductory text message should include (1) providing an overview of STAND, (2) using visuals or links for those who would like additional information about STAND, and (3) ensuring that the intervention is responsive and “human-like.” Encapsulating these themes, one student shared:

*My vision of how I would probably prefer it being shown is a human-like text followed with,* “*I’m from so and so, we - I want to, we’re talking about STAND” and your name at the very bottom, and then a picture or a link about STAND, your website, and what you guys stand for, that would then take you to like, a picture of STAND, like the whole name and the acronym, and then a link that will then follow you to, say, your mission’s goal, who you are, and then, if the person responds, they’ll be able to get a message back. It doesn’t have to be immediate, but at least like a human message back acknowledging, “Hey, I noticed you say this, and I respond with this” like a human text message saying, “I read, I actually took the time to read what you sent me and I‘m going to reciprocate.”*

Regarding content, barriers, consensus emerged among students that the intervention should take a personalized approach to addressing key barriers. Overall, students agreed that the intervention should allow options for recipients to select barriers that are most relevant for the individual and provide tailored responses that directly address the selected barrier. Encompassing this sentiment, one student shared:

*I think each point [barrier] would need a different response. The “I can do it on my own” I think there’s a lack of needs there, like, a lack of acknowledging of needs, of their needs being met. Maybe they’re putting other people first. So, maybe a video of maybe why they should put their needs, their mental needs first? They might, other people might still need them, so they might need to take care of those, too, you know.*

### Between Workshop 1 and 2

The research team used the themes drawn from RQAs of the workshop 1 groups to create preliminary, low-fidelity prototypes of text message frames to present to stakeholders at workshop 2 (Multimedia Appendix 1). These prototypes included an introductory message explaining the STAND program and the text messaging intervention goal, a message asking students to select relevant barriers, and personalized text message responses based on the barriers they selected. These prototypes were developed and reviewed in research team meetings. Content and format of the messages was informed by workshop 1 results and through consultation with experts in digital mental health and mental health treatment engagement (authors DC and DCM). Preliminary refinements were made based on internal feedback and prototypes were prepared for presentation to participants in workshop 2. The research team also discussed key questions about the target population, including time points at which this intervention could be delivered within the STAND workflow. In consultation with the larger research team, we began exploring the idea of aligning text message intervention notifications with existing reminder notification triggers for the current STAND system, occurring after 1, 2, and 7 days of disengagement with screening and orientation or intake scheduling process.

### Build: Workshop 2

The goal of the second workshop was to facilitate rapid prototyping by engaging students in translating ideas generated through the design workshops into low-fidelity, iterative prototypes. The format of this workshop included two sections (1) presenting key themes identified in workshops 1 and generating additional feedback, (2) regarding rapid prototyping, presenting text message prototype frames developed between workshop 1 and 2 and generating participant feedback. Key themes extrapolated through RQAs on workshop 2 sessions are described below by section.

### Workshop 2, Section 1: Feedback on Themes From Workshop 1

Participants were presented with a table and quotes listing the key themes related to intervention method, content, and format characterized in workshop 1 analyses (reported above). They were asked to provide any additional feedback, thoughts, or ideas about themes.

No additional notable themes arose in this process, but students elaborated on previously identified themes. For instance, regarding the format theme of short and limited number of messages, students shared their perspective that these messages should not be “too spammy” and that there should be a “stop” option integrated. For example, one student stated:

*I was going to add, I know you said not too many messages already, but like, make sure it’s not spammy. Or also, of course, I assume you’re going to add the like, “Stop in case you don’t want to receive these messages anymore,” but making sure that’s like clear, because I feel sometimes you sign up with your number and then it’s just spam messages, and I feel like that would be a little bit annoying if it were to happen*

### Workshop 2, Section 2: Rapid Prototyping

Next, we presented participants with preliminary low-fidelity prototypes for the text message intervention content in two main sections (1) introduction to the intervention and (2) personalized barrier selection and response. Students provided feedback about the prototypes. Multimedia Appendix 1 depicts exemplars of the preliminary prototypes that were presented to students in Workshop 2, and reviews specific feedback arising for each barrier, with illustrative quotes. The format or delivery themes described also emerged in this section, including (1) providing students with the option to select more than one barrier getting in the way; (2) allowing students to select no barriers, and receive a link to pick up where they left off with the STAND process; and (3) providing an option to speak to a team member throughout the intervention. With regard to speaking to a team member, one student shared, “having the option to speak to someone is good. Again, if you’re able to schedule it, in case the person can’t do it in the moment, having that option there too would be good.” Another stated, “I like the speak to a STAND team member because it shows that there are people you know, who work for STAND, and you know, being accessible. If you have any questions or anything you can reach out to them.”

### Between Workshop 2 and 3

The research team used the feedback drawn from RQAs of the workshop 2 groups to refine low-fidelity prototypes of text message frames to present to stakeholders at workshop 3 groups for further feedback.

### Build: Workshop 3

The goal of the third workshop was to continue the rapid prototyping process by further refining the prototypes. In this group, the facilitator presented updated prototypes to students and generated additional feedback. Themes identified through RQAs on workshop 3 sessions were related to the content of the intervention and are described below. No format themes arose in the workshop 3 analyses.

Regarding content for introduction, students were shown the updated introduction text prototype, including both text introducing STAND and two images illustrating (1) background information about STAND (including information about the screening, treatment, compensation, and sponsors) and (2) the barrier options to select from, and were asked to provide feedback. Students shared overall approval of the updated introductory text prototype. One student expressed:

*I really like the thread because I feel like as a student, if you see like compensation, it’s gonna draw you to question and like, kind of be curious about what the STAND program is about. So, I really like how it has compensation. And you know, if you’re someone who’s looking for mental health, and then you happen to see that they’re giving you compensation, it’s gonna make you wanna go seek these resources out more.*

No themes emerged, recommending further changes to this portion of the intervention.

Regarding content for barriers, students were shown each updated barrier response prototype, with feedback integrated from workshop 2, and expressed general approval and satisfaction with them. No new significant themes regarding feedback for each barrier response emerged, suggesting that the themes across workshops 1‐3 reached a point of saturation and the intervention was ready to be uploaded into the software system for pilot testing.

### Between Workshop 3 and 4

After workshop 3, we began uploading intervention content into the text messaging software system, Mosio. Mosio is a text messaging software frequently used by researchers to send automated text message reminders and content to participants using a HIPAA-compliant platform (Mosio). For the purposes of our study, we created Mosio “storylines” to create automated, bidirectional, text messaging workflows that send out introductory texts to participants and use branching logic to trigger follow-up messages depending on how the user responds. This feature allowed us to create the personalized barrier responses recommended by stakeholders. Our team uploaded content into Mosio and conducted extensive pilot testing, including 3‐5 rounds with three team members, to identify bugs, glitches, and logic errors and remedy them before testing with students in workshop 4.

We also continued discussing implementation context factors and developed a tentative plan to integrate this intervention at existing reminder triggers within the STAND workflow, occurring after days 1, 2, and 7 of disengagement with screening and orientation or intake scheduling process. We consulted with topic experts on engagement within our research team and reviewed the existing literature to determine that these time points were aligned with research recommendations, as well as the current STAND infrastructure.

### Test: Workshop 4

The goal of the fourth workshop was to pilot the intervention once it was uploaded into the Mosio software platform, with a novel group of student users, generate additional feedback, and explore usability factors. Students brought their cell phones to the workshop and were asked to read and respond to messages as if they were a real user receiving them. They were sent the introductory message and encouraged to select whichever barriers they wanted to. Overall, students shared positive perceptions of text message content, and concerns and suggestions related to formatting issues emerged. Students were also asked to rate usability factors at the end of the workshop. Similar themes about format and content from workshops 1‐3 emerged in workshop 4.

Regarding format or delivery of images, memes, or visuals, students expressed that the memes and images used throughout the intervention were entertaining and engaging. One student shared, “I like the meme that is sent…I thought that was kind of funny.” Another student said, “The little picture things, the little memes actually, really adorable and more engaging.” They also expressed positive regard for the infographics, emphasizing that they were clear, provided helpful information, and were easy to digest. For instance, one student stated, “I think it was good, straightforward, short.” Another shared, “The flyer was helpful, because when I first started I was like...that’s kind of like in the dark I didn’t know what to expect, but that broke it down pretty well….It’s short, and it broke it down in different colors, which it’s helpful for me.” Students also expressed that interspersing the images with text makes the intervention less daunting, for instance, one shared, “I think that the image helps because it makes it less overwhelming.”

Regarding format or delivery of short text and limited messages, as with previous workshops, students expressed some concern that messages were still too text-heavy. For example, one student stated, “I actually felt intimidated to immediately get like 5 messages like Boom! Boom! And like, the 1st thing I see is like all these words, and I just feel like, Oh, my God! It’s a lot!” Another student said:

*I get a lot of text messages like this on my phone. especially with this, with the election like Nonstop. And so it’s a lot of spam. I don’t know how they get my number, but I get a lot of it. so maybe I don’t know. I think for me. If it was at least the initial text was shorter and maybe some way for it to stand out so that I know it’s not spam.*

Students also shared concerns that some text might be buried or overlooked. For example, the introductory message with the barrier options instructed students to select one barrier at a time. Students recommended further highlighting guidance to select one barrier at a time, noting that this instruction could be easily missed. One student shared:

*Yeah, I think specifically, the part where it says you can select another one at a later time is what got me. I didn’t. I completely missed that. So I like selected multiple. And then I got the same message like I wasn’t able to receive the message.*

Regarding content, overall, students expressed positive reactions to the content of the text messages, including both the introduction and personalized barriers. One student shared about the intervention as a whole: “It’s concise, and I think it addresses most issues that people can have.*”* Students also shared that barriers were personally relevant, and tailored content helped address them. For example, in response to the “I am concerned about privacy and confidentiality” barrier content, one student shared, “I think it’s super helpful. This was one of the problems that I had…finding a space. So I’d walk around campus looking for like an empty space. So I kind of bummed out that I didn’t get to benefit from that.” Students also liked that the intervention allowed for them to opt to speak to a STAND team member if desired. One student said, “you get a text message saying that they’ll reach out to you within 24 hours, I figured that that’s what I would take if I was, you know, really running it through. So, I thought that was cool. All I have to do now is, look out for the message.” Another student shared, “I like that somebody would get back to me.”

### Usability Ratings

After completing the pilot text message testing process, participants were asked to complete usability ratings on a number of domains to measure their overall experience with the intervention. Mean ratings on usability factors ranged from 3.88 (SD 0.64) to 4.25 (SD 0.46) of 5 (Table 4).

**Table 4.** Usability ratings.

Usability domain	Values, mean (SD)
Satisfaction	4.25 (0.46)
Helpfulness	4 (0.53)
Attention grabbing	3.875 (0.83)
Readability	4 (0.76)
Comprehension	3.875 (0.64)
Likelihood of starting STAND^a^	4.125 (0.64)

a STAND: Screening and Treatment for Anxiety and Depression.

### After Workshop 4

Upon completion of workshop 4 analyses, we continued making iterative refinements to the text messaging prototype in response to student concerns about text messages being too text-heavy and “spammy.” We reduced text length and the number of messages, and continued revising visuals in an effort to make them further stand out and engage students. A final presentation of the text message flow and example content is illustrated in Multimedia Appendix 2.

## Discussion

### Key Findings

This study describes the process of co-designing a text messaging intervention to address barriers to starting a mental health screening and treatment program, STAND, for CC students. Given the high mental health needs and low service engagement among CC students, we sought to create an intervention in direct collaboration with the target user group in order to increase its likelihood of success when implemented. Further, given that CC students represent an overlooked and underserved population, we were intentional in our use of a cocreation process that put student voices at the forefront of our intervention design. We used implementation science and human-centered design principles within the context of a multistep, iterative prototyping research process to engage students in a series of cocreation workshops culminating in a text messaging microintervention prototype. The following section outlines key themes, lessons learned, and implications of this study. Findings reflect preliminary evidence and should be interpreted with caution, given that this project sample was small, drawn from one setting, and participants had some prior exposure to the mental health intervention.

Key themes derived from the co-design workshop series emphasized that text messaging is perceived as an acceptable, helpful, and feasible method of intervention delivery for students. Results also emphasized design considerations that arose in domains of content (eg, what information does the intervention contain?) and format or delivery (eg, how is the intervention information delivered?). With regard to content, students favored an approach that provides a broad introduction to the overall mental health program and context (STAND), followed by opportunities to personalize the intervention to their specific needs by selecting barriers of most relevance to the individual. Throughout, students expressed preferences for personalization, for more knowledge about STAND, testimonies of other students, reminders that they are not alone, and expressing understanding of student lives and needs. Format recommendations included integrating multimedia strategies to deliver content (eg, text, images, memes, infographics, videos, and options to speak to a team member). In addition, students expressed that messages should be short, low-density, and not too frequent or intrusive. These themes arose repeatedly across workshops in content and format domains, highlighting the saliency of these perspectives among students.

### DDBT Model Application

Our process also demonstrates novel extensions of the DDBT model in two ways. First, we used this model to co-design a digital “microintervention” within the context of a larger multitiered screening and intervention program rollout. Second, we applied this model within a CC population and setting. Many former applications of DDBT have focused on redesigning and adapting existing interventions and creating strategies to improve the implementation of evidence-based interventions in community settings. Some DDBT applications have also focused on harnessing digital tools to deliver mental health interventions [29]. Our study uniquely focused on using the DDBT model to create a digital “microintervention” with a focus on creating attitudinal change with potential to lead to behavioral change within the context of a larger clinical research and mental health service initiative. While DDBT was created for use in low-resource contexts, no known studies have focused on its application with CC students. This study illustrates that the DDBT framework facilitated strong engagement of CC students in a co-design process and was a helpful and acceptable guiding model for research and cocreation activities.

The DDBT model focuses on usability as a key outcome. Our results illustrated that use of the DDBT model for co-design in this context was associated with positive usability outcomes. Participant quantitative ratings of the text message intervention demonstrated above-average satisfaction, helpfulness, and utility of the intervention, and qualitative themes and quotes highlighted satisfaction, positive feedback, and acceptability of the intervention. While these results were positive, this pilot sample was very small and may lack generalizability. These findings should be viewed as early and encouraging, and the degree to which they are reflective of the broader population remains unknown. Next steps involve pilot testing of the intervention to explore preliminary effectiveness in improving uptake of the STAND program among CC students. This future work will provide a more rigorous test of the success of this intervention.

### Broader Implications

Findings from this project also have implications that can inform intervention, implementation, and policy in the CC context more broadly. First, our findings highlighted the importance to students of personalized approaches. Tailoring of engagement interventions to meet the specific needs, preferences, and experiences of individual students appears to be a compelling and desirable strategy in the CC student mental health landscape. Second, our findings emphasize that technology-driven pathways to engagement, including text messaging, are acceptable and positively viewed by CC students. These approaches have strong potential to address key barriers to treatment and deliver important mental health information and psychoeducation to students. Third, students in this study repeatedly emphasized their desire for transparency and information about mental health services or programs they are signing up for. This point may have broader significance beyond text messaging and engagement, illustrating the importance of clearly articulating and widely advertising descriptions, goals, and processes associated with mental health programs and services.

### Lessons Learned

Our process also revealed many challenges and complexities arising across co-design activities and procedures. In the co-design process, the two primary parties involved are users and researchers, both of whom engage in the process with different perspectives, biases, goals, and backgrounds [31]. The varying perspectives of these parties facilitate a rich and nuanced process, but they can also pose challenges due to power imbalances and constraints such as limited connection, time, and funding [31]. In our process, we regularly encountered scenarios in which differences in perspective, skills, and contexts of researchers and students were at the forefront. We next describe some of these situations and how we approached them with principles of joint inquiry and imagination [32].

One key tension that we grappled with repeatedly was how to strike a balance between imagination and feasibility. While we strived to prioritize and implement all student perspectives and ideas, we also set out to create an intervention that could be feasibly implemented within the constraints of an existing system, software, and framework. We wanted students to “paint outside the lines,” but also realized that we would need to “frame the canvas,” to ensure feasibility. When discussing this tension as a research team, we concluded that prioritizing student ideas and voices was most important. Thus, when students proposed ideas that our team perceived as limited in feasibility, we responded by (1) carefully examining alternative, creative possibilities for bringing student ideas to life, (2) validating and reinforcing student ideas, and (3) acknowledging system constraints when they posed significant barriers. We believe that this approach allowed students to feel heard and understood, while also enabling our team to be pragmatic and transparent about limitations.

A related topic that arose frequently was students’ desire for intervention personalization, which the research team grappled with, given the limitations of our software system and context. In one example, some students suggested that the intervention should allow students to write in their own barriers and suggested having a human respond to these in real-time. However, our software was unable to handle participant open responses, and we had insufficient human personnel capacity to respond to personalized texts in real time. We ultimately prioritized as much personalization as possible within our constraints. For example, we allowed students to select “something else is getting in the way” as a barrier and provided options to speak to a team member via phone about this. We ensured that student recommendations were captured and documented for potential future iterations, should more funding and flexibility become available.

Another topic we wrestled with was responding to feedback about the STAND program at large, as opposed to our text messaging intervention in particular. For example, some students recommended reducing the length of the screening survey to decrease participant burden. These recommendations for the broader STAND program were communicated to the research team. However, our co-design focus was on a specific microintervention within this larger context, and we were limited in our ability to adjust the larger system. We approached these suggestions by validating and reinforcing participant feedback, acknowledging limitations due to research infrastructure, and communicating feedback to the team leaders. This tension emphasizes the need for an even more comprehensive approach to co-design that spans all components of a system and accounts for multiple, often competing, interests and goals. Further, the use of new technological innovations, such as artificial intelligence (AI), may have the capacity to improve intervention personalization and reduce technology constraints. Future research that leverages AI for this purpose represents an essential next step.

### Limitations

This study has several notable limitations. First, our sample was relatively small; thus, findings may not be generalizable to all students. Second, due to limitations regarding data collection, we were unable to ascertain the degree to which our sample was representative of the larger STAND sample. However, because a large proportion of our sample was drawn from a pool of students who started or completed the STAND screening and orientation, our workshop participants at least in part reflect the larger population STAND serves. Although overall student perceptions and ratings of the intervention were positive in workshop 4, they provided some additional constructive feedback reiterating concerns about the number and density of text messages. While we attempted to address these changes after the workshop, additional user feedback should be gathered from students in future studies, and the intervention should continue to be refined. Last, because our study required participants to have some context and understanding of the STAND program, our sample may not accurately reflect the beliefs and experiences of students without any previous exposure to STAND or mental health. Collecting data to improve our understanding of students’ viewpoints who have even more limited mental health exposure is needed to fully address barriers to engagement in our intervention development.

### Conclusions

Overall, this study demonstrates that CC student stakeholders can be engaged in a co-design process to create a text messaging intervention focused on improving initiation of mental health services. This study is among the first to apply co-design methods and the DDBT framework to a high-need, low-resource CC student population and to the design of a text messaging intervention. These community-partnered efforts are essential to creating effective, responsive interventions and simultaneously building the mutual trust, respect, and understanding needed for future collaboration efforts. Although the sample was small, the usability outcomes found in this study were encouraging, emphasizing potential acceptability and satisfaction with the text messaging prototype. Future research will continue the iterative design process and test the preliminary effectiveness of this intervention in a larger context, with the goal of increasing engagement in STAND services. We plan to embed this intervention within the STAND screening flow and conduct a pilot open trial in which a subset of participants who drop off along the STAND engagement process will receive the text message intervention. We hypothesize that the text messaging intervention will be effective in improving rates of STAND uptake because it was designed in partnership with CC students using co-design methods and was rated as highly usable in this foundational study.

## Supplementary material

10.2196/84640 Multimedia Appendix 1 Text message prototypes, themes, and quotes.

10.2196/84640 Multimedia Appendix 2 Final text message intervention flow and content presentation.

## Abbreviations

CC: community college
DDBT: Discover, Design, Build, and Test
ELAC: East Los Angeles College
HCD: human or user-centered design
HIPAA: Health Insurance Portability and Accountability Act
RQA: rapid qualitative analysis
STAND: Screening and Treatment for Anxiety and Depression
