# Supplementary material for: Developing a Text Messaging Intervention to Increase Uptake of the Screening and Treatment for Anxiety and Depression Program Among Community College Students: Formative Study Using a Human-Centered Design Approach
Source: JMIR Form Res. 2026 Jul 21;10:e84640. doi: 10.2196/84640 (PMC13387416; doi:10.2196/84640)
Supplement: Multimedia Appendix 1 [file formative-v10-e84640-s001.docx]

| **Text message component** | **Barrier label** | **Content** | **Format** | **Quotes** | **Prototypes** |
| --- | --- | --- | --- | --- | --- |
| Introductory text message | N/A | Provide information about what messages are for and what the STAND program is.  Provide chance to select barrier(s) most relevant for individual.  Include STOP option. | Images  Text |  | 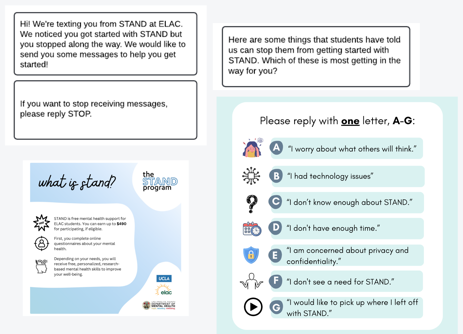 |
| Barrier: stigma | I worry about what others will think. | Provide reminders that you are not alone.  Provide encouragement that you can overcome stigma and get help. | Images  Text with information | “*Personally, for me, I really like the image and text, the “You're not alone,” like, well, I really like the aesthetic and like how eye-pleasing the message is. So, I think that's the first thing that pops- like catches my attention, that “you're not alone in this” and that it’s colorful, but it's not too bright, too much.”* | 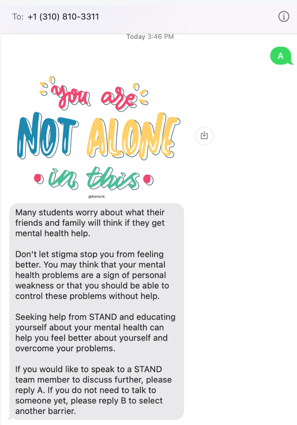 |
| Barrier: technology issues | I had technology issues | Provide information about common technology challenges | Meme  Text with options (speak to team member, view image with more information) | *“I think they'll be really effective. It feels like you're not talking to a bot, it feels like you're talking with someone- it's like your friend sending you a meme, and you just start laughing, and it's just like something like that. It feels like I'm texting a real person.”* | 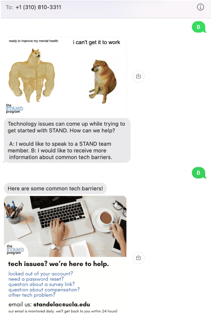 |
| Barrier: Lack of knowledge about STAND | I don’t know enough about STAND. | Provide information about STAND. | Text with options (video, speak to team member, view image with more info) | *“Yeah, I feel like it shouldn't just be like a video explaining STAND, it should be, you know, just like some text messages, also explaining what STAND is. You don't have to speak, you don't have to watch, maybe just some- a little like brief summary.”* | 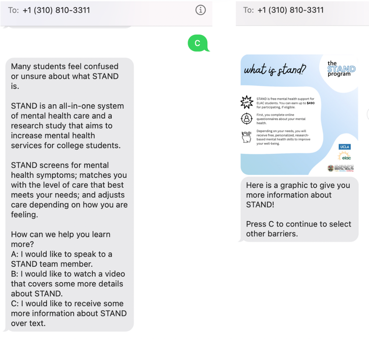 |
| Barrier: Not enough time or too busy for STAND | I don’t have enough time. | Validate student busy schedules.  Provide options for reminders and information about how long STAND activities take. | Meme  Text with options (schedule reminder, speak to team member, view image with information) | *“I know personally, I rely on my reminders app. So, having those reminders really benefits the students to remind themselves to actually log in, because if I don't do that or write it on a sticky note, I will not come back to something.”*  “*I like the doggie on fire. Yeah, I've seen that one around, as well as the one on top. Again, I think because they're on social media a lot”.*  “*We could include the steps we could put like how much time each step takes, so that they know how much time they might need to kind of carve out of their schedule for it.”* | 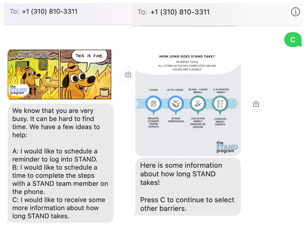 |
| Barrier: Concerns about privacy and confidentiality | I have concerns about privacy and confidentiality. | Validate student privacy/confidentiality concerns.  Provide options for room scheduling and information about STAND data privacy and confidentiality. | Text with options (book private room, speak to team member, image with more information) | “*I think this has a good variation. I feel like being able to book directly from, I assume the text message, we would send a link or something like that would be really good because typically when they're like, “Oh, would you like to do this? Go to this website” I never go to the website. So, if you're able to send a link directly, I think that would be good.”* | 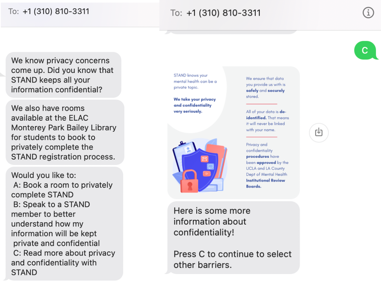 |
| Barrier: Lack of perceived need for STAND | I’m not sure if I need STAND. | Provide information about number of students who have received STAND.  Let students know that they can stop if not working.  Remind that mental health matters. | Image + text with information and option to speak to team member | “*I was thinking more like addressing the issue of need and maybe providing data of like why a student needs it, and how going to the program helps overall—their whole life, because if someone responds that they don't see a need. I feel like there's other issues or thoughts behind that”.* | 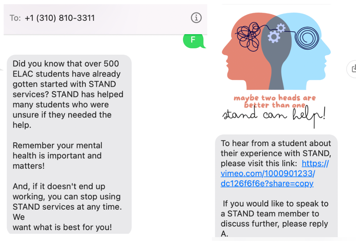 |
