# Supplementary material for: Developing a Text Messaging Intervention to Increase Uptake of the Screening and Treatment for Anxiety and Depression Program Among Community College Students: Formative Study Using a Human-Centered Design Approach
Source: JMIR Form Res. 2026 Jul 21;10:e84640. doi: 10.2196/84640 (PMC13387416; doi:10.2196/84640)
Supplement: Multimedia Appendix 2 [file formative-v10-e84640-s002.pdf]

Final Text Message Intervention Flow & Content Presentation

This appendix illustrates screen shots to overview the final text message intervention flow and content.

The basic flow includes three main sections:

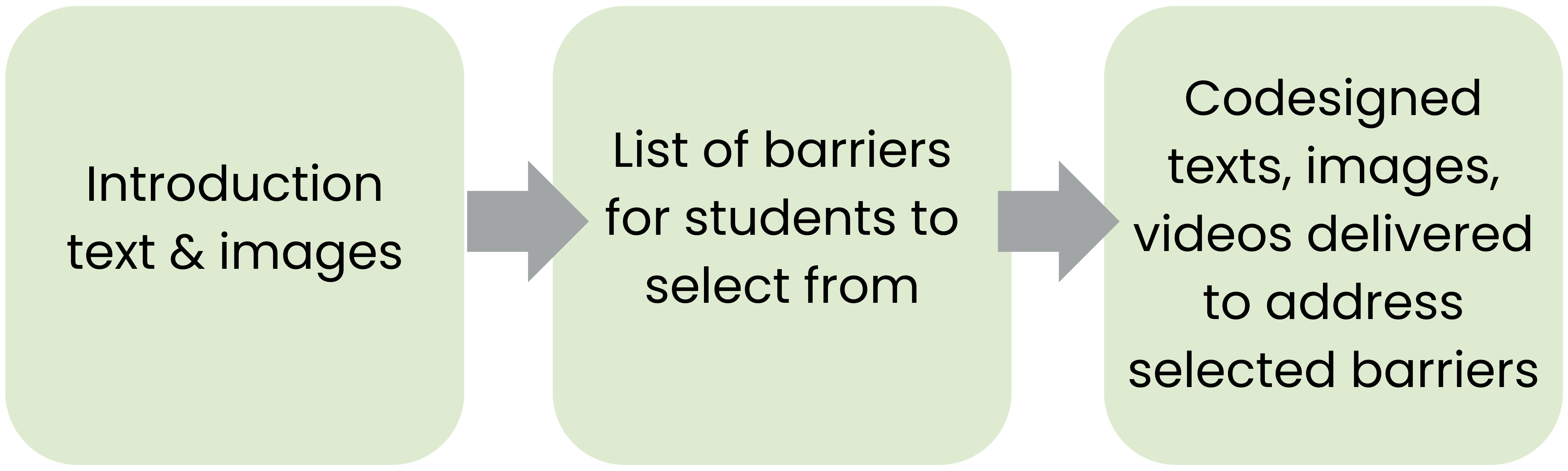

Final Text Message Intervention Flow & Content Presentation

Introduction Text

To: +1 (310) 810-3311

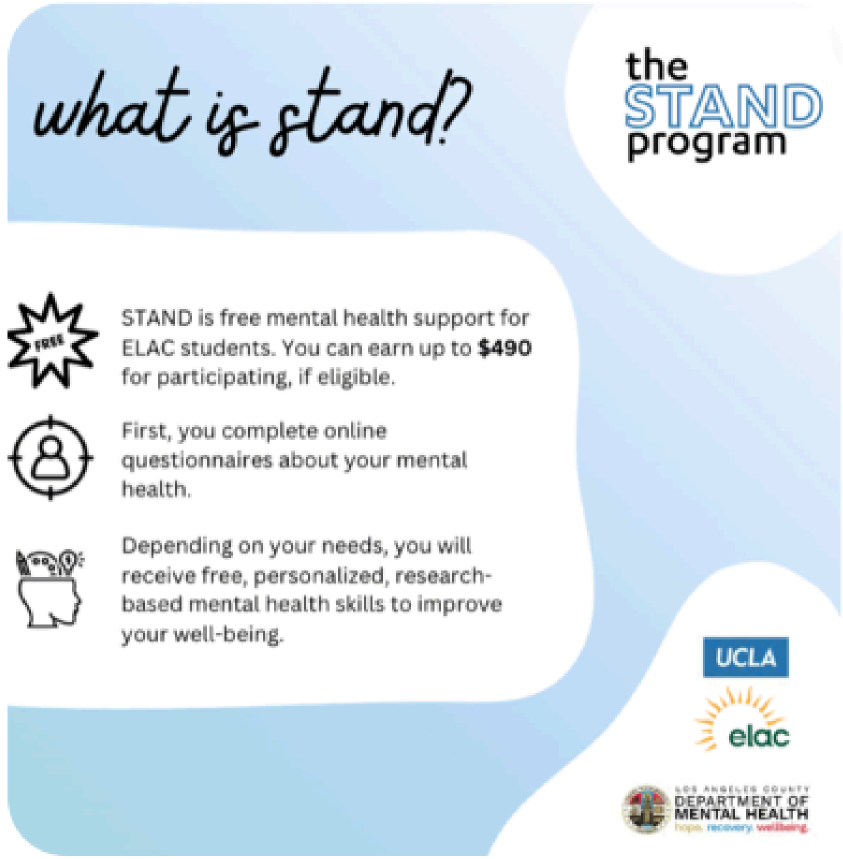

Hi! We're texting you from STAND at ELAC. We noticed you got started with STAND but you stopped along the way. We would like to send you some messages to help you get started!

Barriers Selection

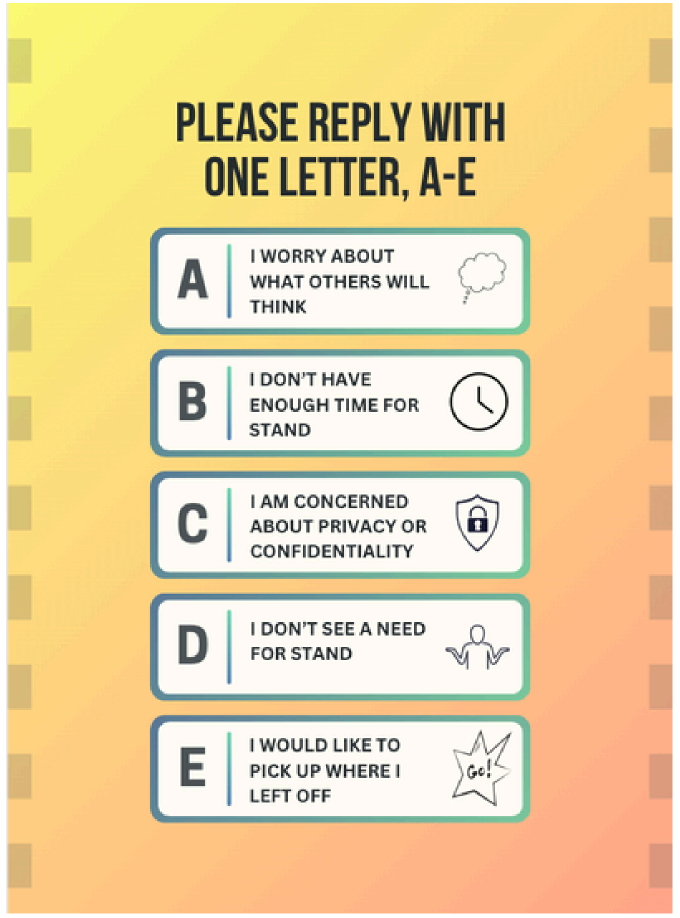

Here are some things that students have told us can stop them from getting started with STAND. Which of these is most getting in the way for you?

Final Text Message Intervention Flow & Content Presentation

Barriers Selection

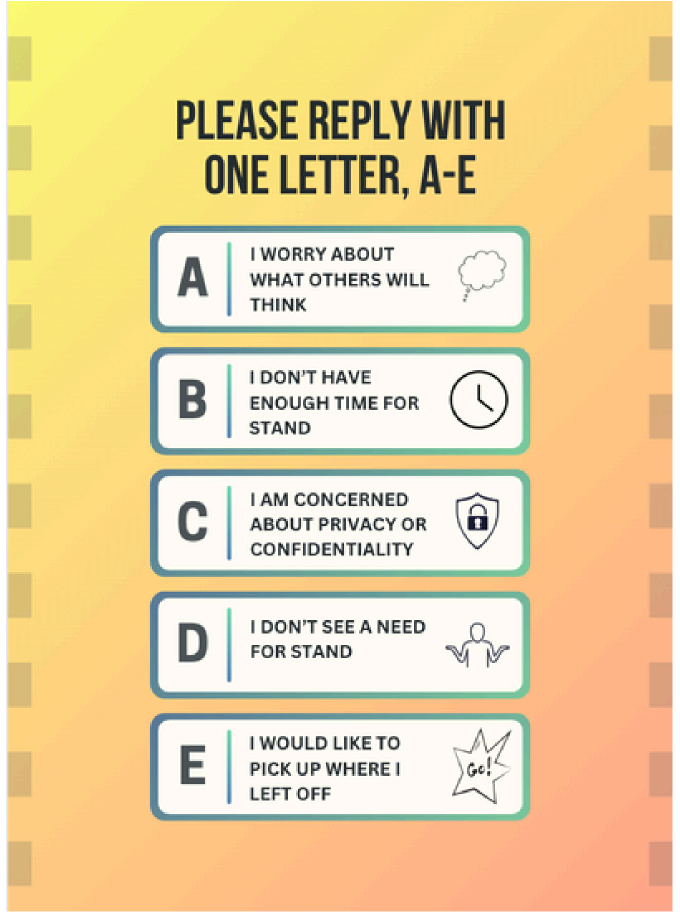

Here are some things that students have told us can stop them from getting started with STAND. Which of these is most getting in the way for you?

Here, student replies "A" to select "I worry about what others will think"

To: +1 (310) 810-3311

Today 3:46 PM

A

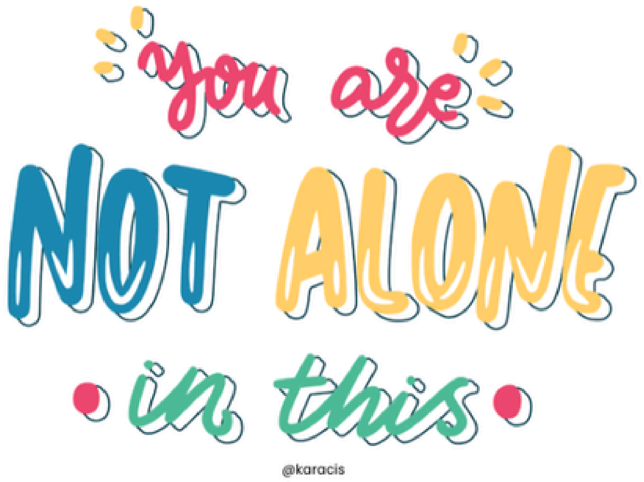

Many students worry about what their friends and family will think if they get mental health help.

Don't let stigma stop you from feeling better. You may think that your mental health problems are a sign of personal weakness or that you should be able to control these problems without help.

Seeking help from STAND and educating yourself about your mental health can help you feel better about yourself and overcome your problems.

If you would like to speak to a STAND team member to discuss further, please reply A. If you do not need to talk to someone yet, please reply B to select another barrier.

Final Text Message Intervention Flow & Content Presentation

Here, student replies "B" to select "I don't have enough time for STAND"

To: +1 (310) 810-3311

B

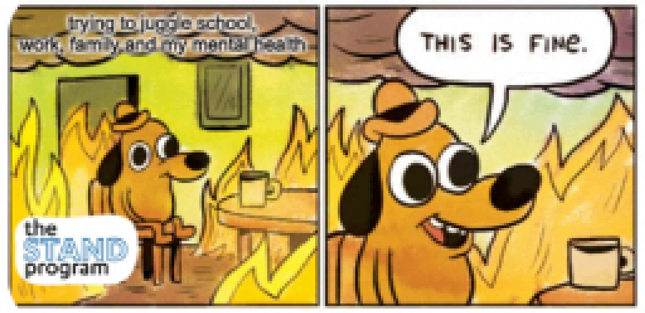

We know that you are very busy. It can be hard to find time. We have a few ideas to help:

A: I would like to schedule a reminder to log into STAND.
B: I would like to schedule a time to complete the steps with a STAND team member on the phone.
C: I would like to receive some more information about how long STAND takes.

Then, they select "C" from the menu of options shown, and receive:

To: +1 (310) 810-3311

C

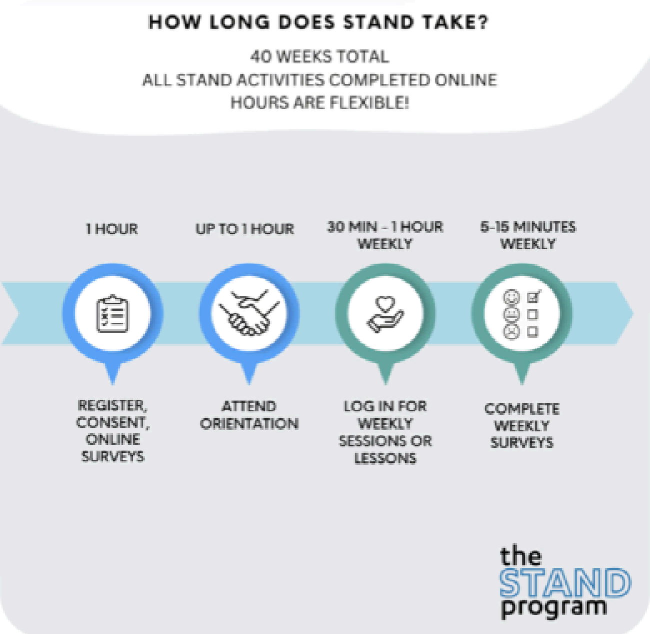

Here is some information about how long STAND takes!

Press C to continue to select other barriers.

Final Text Message Intervention Flow & Content Presentation

Here, student replies "D" to select "I don't see a need for STAND"

To: +1 (310) 810-3311

D

Did you know that over 500 ELAC students have already gotten started with STAND services? STAND has helped many students who were unsure if they needed the help.

Remember your mental health is important and matters!

And, if it doesn't end up working, you can stop using STAND services at any time. We
want what is best for you!

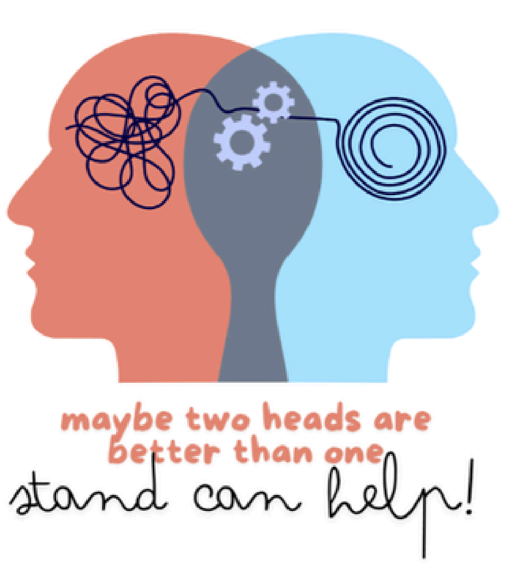

To hear from a student about their experience with STAND, please visit this link: https://vimeo.com/1000901233/dc126f6f6e?share=copy

If you would like to speak to a STAND team member to discuss further, please reply A.
